# Membrane Protein Biogenesis in Ffh- or FtsY-Depleted *Escherichia coli*

**DOI:** 10.1371/journal.pone.0009130

**Published:** 2010-02-09

**Authors:** Ido Yosef, Elena S. Bochkareva, Julia Adler, Eitan Bibi

**Affiliations:** Department of Biological Chemistry, Weizmann Institute of Science, Rehovot, Israel; Baylor College of Medicine, United States of America

## Abstract

**Background:**

The *Escherichia coli* version of the mammalian signal recognition particle (SRP) system is required for biogenesis of membrane proteins and contains two essential proteins: the SRP subunit Ffh and the SRP-receptor FtsY. Scattered *in vivo* studies have raised the possibility that expression of membrane proteins is inhibited in cells depleted of FtsY, whereas Ffh-depletion only affects their assembly. These differential results are surprising in light of the proposed model that FtsY and Ffh play a role in the same pathway of ribosome targeting to the membrane. Therefore, we decided to evaluate these unexpected results systematically.

**Methodology/Principal Findings:**

We characterized the following aspects of membrane protein biogenesis under conditions of either FtsY- or Ffh-depletion: (i) Protein expression, stability and localization; (ii) mRNA levels; (iii) folding and activity. With FtsY, we show that it is specifically required for expression of membrane proteins. Since no changes in mRNA levels or membrane protein stability were detected in cells depleted of FtsY, we propose that its depletion may lead to specific inhibition of translation of membrane proteins. Surprisingly, although FtsY and Ffh function in the same pathway, depletion of Ffh did not affect membrane protein expression or localization.

**Conclusions:**

Our results suggest that indeed, while FtsY-depletion affects earlier steps in the pathway (possibly translation), Ffh-depletion disrupts membrane protein biogenesis later during the targeting pathway by preventing their functional assembly in the membrane.

## Introduction

*E. coli* proteins Ffh and FtsY are homologues of the mammalian SRP54 protein and the SRP-receptor α-subunit (SRα), respectively [1], [2]. The bacterial SRP system plays important role in the biosynthetic pathway of many inner membrane proteins [3], [4], as shown both by genetic studies [5]–[10], and using *in vitro* systems [e.g.], [11,12]. *In vitro*, FtsY displaces the SRP from nascent membrane proteins [13] and therefore it is assumed that in analogy to the mammalian SRP, the *E. coli* SRP (Ffh and 4.5S RNA) functions upstream of the SRP-receptor, during the targeting of ribosomes translating membrane proteins to the cytoplasmic membrane. Accordingly, it is anticipated that interrupting the function of either of these essential components (Ffh or FtsY) would lead to similar phenotypes regarding ribosome targeting and membrane protein biogenesis. In order to better understand the involvement of FtsY and Ffh in membrane protein biogenesis *in vivo*, we have studied the expression, localization and assembly of model membrane proteins in cells depleted of either of these proteins. We show that only FtsY-depletion leads to specific inhibition of membrane protein synthesis, whereas Ffh-depletion has a marked effect on their functional assembly in the membrane.

These results, in combination with previous studies, support an alternative order of events during the SRP pathway in *E. coli* [3]. FtsY-depletion inhibits ribosome targeting [14] and concomitantly leads to specific inhibition of membrane protein synthesis. In contrast, Ffh-depletion affects the pathway downstream, possibly at the stage where ribosomes nascent chain complexes are transferred and assembled on the translocon.

## Results

### Expression of Membrane Proteins in FtsY- or Ffh-Depleted Cells

**Figure 1:**
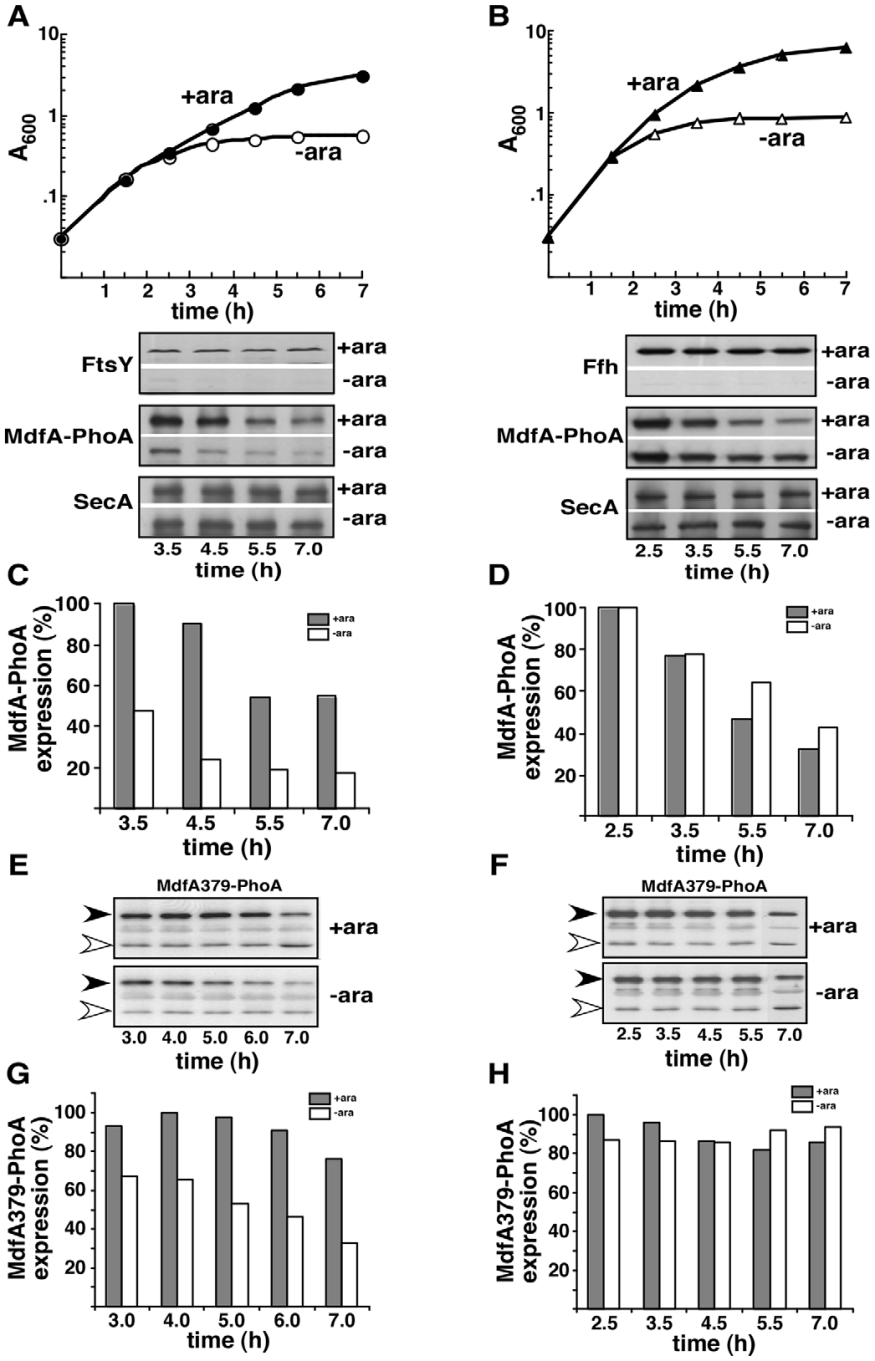
Different effect of FtsY- or Ffh-depletion on expression of MdfA-PhoA and MdfA379-PhoA. *E. coli* FJP10 cells (left panels) or WAM121 (right panels), harboring the arabinose inducible *ftsY* or *ffh* genes, respectively, were transformed with plasmids expressing MdfA-PhoA (A-D) or MdfA379-PhoA (E-H), and grown with or without arabinose (A and B, upper panels). Extracts of samples withdrawn at the indicated times were examined by Western blotting using anti-FtsY, anti-Ffh, anti-PhoA and Anti-SecA antibodies (A-B, lower panels and E-F). In all cases, 10 µg of total protein were analyzed. In E and F, the positions of the MdfA379-PhoA and free (likely cleaved) PhoA are marked by filled and empty arrowhead, respectively. Expression of MdfA-PhoA (C, D) and MdfA379-PhoA (G, H) were quantified by densitometry and presented by dark columns for non-depleted samples and light dotted columns for depleted samples. The experiments were repeated 2–3 times and the results shown are representative and standard deviation did not exceed 15%.

The multidrug transporter MdfA [15] was chosen as a model for the following comparative studies. MdfA is an integral membrane protein with 12 hydrophobic trans-membrane helices (TMs) [16]. In order to facilitate identification of the protein by Western blotting and for further analysis of its membrane assembly (see later), we used a signal peptide-deleted version of alkaline phosphatase as a C-terminal tag. Two hybrids were studied [both described in ref 16]: (i) Full-length MdfA-PhoA, in which alkaline phosphatase was fused to the cytoplasmic C-terminus of MdfA, and (ii) a hybrid in which alkaline phosphatase was fused to the periplasmic loop after TM 11 (MdfA379-PhoA). The hybrids were expressed in *E. coli* strains that enable arabinose-dependent depletion of either FtsY (FJP10) [17] or Ffh (WAM121) [6]. Figure 1 (A and B) shows typical growth curves of these strains transformed with plasmids encoding either of the two MdfA-PhoA hybrids in the presence or absence of arabinose. Effective depletion of FtsY (Fig. 1A, lower panel) or Ffh (Fig. 1B, lower panel) was achieved after 2.5–3.5 h. As shown, MdfA-PhoA expression decreases with time in all cases (Fig. 1A and B, lower panels). However, whereas the expression in cells +/− Ffh is similar throughout the experiment (Fig. 1D), the expression of MdfA-PhoA in cells depleted of FtsY is drastically inhibited compared to cells expressing the receptor (Fig. 1C). In all cases, the expression of SecA, a soluble protein, remained almost constant throughout the experiment (Fig. 1A and B, lower panels). Unlike with MdfA-PhoA, the second hybrid MdfA379-PhoA exhibited a relatively stable expression with time in arabinose-induced cells (Fig. 1E and G, F and H). Nevertheless, as with MdfA-PhoA a substantial decrease in the expression of MdfA379-PhoA was observed only under FtsY-depletion conditions compared with cells depleted of Ffh (Figure 1, compare G with H). These results show that the expression of the same membrane proteins is affected differently in Ffh- compared to FtsY-depleted cells. Importantly, as shown here with SecA and previously with LacZ or β-lactamase [7], EF-G, CAT, YjeQ, SmpB, and UspG (data not shown), the inhibitory effect of FtsY-depletion is specific for membrane proteins.

### Membrane Protein Biogenesis in FtsY-Depleted Cells

**Figure 2:**
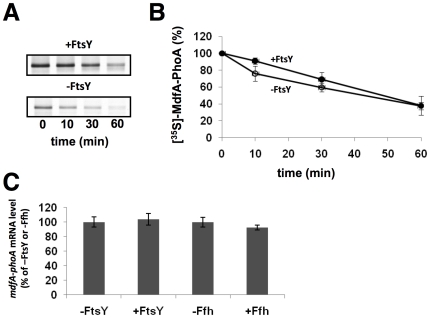
Effect of FtsY-depletion on the stability of MdfA-PhoA and on the amount of its coding mRNA. *E. coli* FJP10 harboring plasmid pT7-5/MdfA-PhoA was grown with and without arabinose in 2YT broth for 4.5 h and transferred into M9 minimal broth for [^35^S]-methionine labeling. (A) Samples taken at the indicated time points after the chase with cold methionine were analyzed by immunoprecipitation using anti-alkaline phosphatase antibodies. (B) The amount of labeled MdfA-PhoA was quantified by densitometry and the average of three independent assays is shown, with error bars representing standard deviations. (C) The expression levels of MdfA-PhoA mRNA were analyzed using real time PCR and normalized to that of ribosomal 16S rRNA. Samples without arabinose (-FtsY and –Ffh) were set to 100%. The average of three separate assays is shown, with error bars representing standard deviations.

Theoretically, various stages of expression could be affected under FtsY-depletion conditions, several of which were examined here. First, we explored the possibility that membrane proteins might be proteolyzed via a quality control mechanism that prevents their accumulation and aggregation in the cytoplasm [18]. This was accomplished by testing the stability of MdfA-PhoA using pulse and chase experiments with FtsY-depleted or non-depleted cells. After 2 min of pulse-labeling with [^35^S] methionine and chase with cold methionine, samples were subjected to immunoprecipitation, SDS-PAGE, and autoradiography. As shown in Fig. 2A, and in agreement with the expression results (Fig. 1), the amount of MdfA-PhoA expressed during the pulse-labeling in FtsY-depleted cells is significantly lower than that observed in samples taken from non-depleted cells. However, no difference in MdfA-PhoA stability was observed between samples prepared from depleted or non-depleted cultures even after 60 min of chase (Fig. 2B). Identical results were obtained previously with another membrane protein, LacY (Andrei Seluanov and E.B., unpublished), suggesting that post-translational proteolysis does not account for the low expression of membrane proteins in FtsY-depleted cells.

Next, we investigated the possibility that the observed low expression of membrane proteins in FtsY-depleted cells reflects an impaired transcription or mRNA degradation. This was approached by measuring the amount of mRNA encoding the test membrane protein in FtsY-depleted or non-depleted cells by quantitative RT-PCR. Total RNA was subjected to RT-PCR using synthetic oligonucleotides for the coding reagion of *mdfA* and the ribosomal RNA 16S as control (see Methods). The results show that the amount of the MdfA encoding mRNA is similar in all the 4 samples obtained from non-depleted or depleted (Ffh, or FtsY) cells (Fig. 2C). In conclusion, since no decrease was detected in the stability of membrane proteins or in the amount of their encoding mRNAs, these studies suggest that the decreased expression of membrane proteins in FtsY-depleted cells might be due to inhibition of translation.

### Membrane Protein Biogenesis in Ffh-Depleted Cells

**Figure 3:**
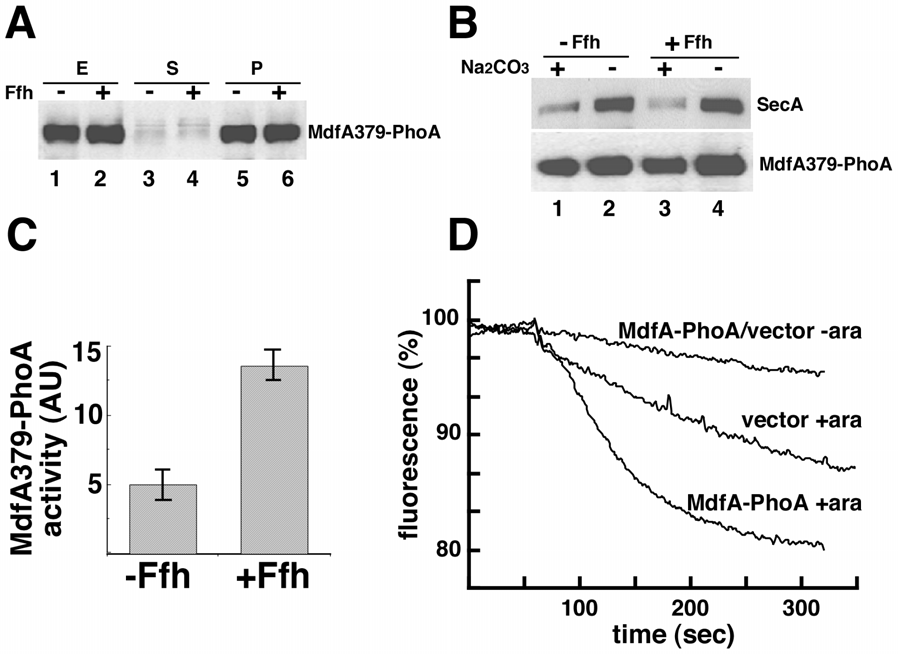
Effect of Ffh-depletion on the localization and functional assembly of MdfA. (A) Extracts from Ffh-depleted or non-depleted WAM121 cells harboring pT7-5/Mdf379-PhoA were fractionated (E, extracts; S, supernatant; P, pellets). Examination of MdfA379-PhoA in the different fractions was accomplished by Western blotting. (B) Membranes isolated by floatation were collected by ultracentrifugation without and with pre-treatment with 0.2 M Na_2_CO_3_ (pH 11) and analyzed by Western blotting using anti-PhoA and Anti-SecA antibodies. (C) The alkaline phosphatase activity of MdfA379-PhoA in WAM121 cells is presented as the rate of p-nitrophenyl phosphate hydrolysis. The experiments were repeated 3 times and error bars indicate standard deviations. (C) Efflux activity of MdfA in Ffh-depleted or non-depleted cells was measured by following EtBr fluorescence. The experiments were repeated at least 3 times, and the results shown are representative.

Our results showed that membrane protein expression level is not affected by Ffh-depletion. To characterize further the fate of MdfA379-PhoA and MdfA-PhoA in Ffh-depleted cells, we examined their localization, assembly, and biological activity under these conditions. Initially, the intracellular localization of MdfA379-PhoA was studied by fractionation experiments and Western blotting. Extracts prepared from Ffh-depleted or non-depleted cells (Fig. 3A, lanes 1 and 2) were ultracentrifuged and no MdfA379-PhoA was detected in the supernatant (Fig. 3A, lanes 3 and 4). Next, the pellets (Fig. 3A, lanes 5 and 6) were resuspended and membranes were purified by floatation (Fig. 3B). To examine membrane integration of MdfA379-PhoA, the purified membranes were washed by sodium carbonate (alkaline pH). Figure 3B shows that the peripheral membrane protein SecA is removed efficiently from the membranes under high pH conditions (Fig. 3B, upper panel, compare lanes 1 and 3 with lanes 2 and 4), whereas a large fraction of MdfA379-PhoA remains associated with the membrane, regardless of whether Ffh was present or absent (Fig. 3B, lower panel, compare lanes 1 and 3 with lanes 2 and 4). The results suggest that MdfA379-PhoA is targeted and inserted into the membrane under these conditions. We further asked whether the membrane integrated MdfA379-PhoA hybrid acquires a similar folded state in Ffh-depleted and non-depleted cells. To this end, we examined its membrane topology. In wild-type cells, the alkaline phosphatase moiety of this hybrid is translocated to the periplasmic space where it exhibits relatively high alkaline phosphatase activity [16]. Measurements of the specific alkaline phosphatase activities of MdfA379-phoA in both strains revealed that the hybrid was significantly less active in the Ffh-depleted cells than in non-depleted cells (Fig. 3C), indicating improper topology. Finally, we examined the drug transport activity of MdfA-PhoA utilizing EtBr transport assays [**19**] **.** As a representative experiment, Figure 3D shows that whereas MdfA-PhoA exhibits high EtBr-efflux activity (decreased fluorescence) in non-depleted cells, it has no transport activity in Ffh-depleted cells. Interestingly, control cells harboring plain vector also exhibit better efflux activity when induced for Ffh expression, suggesting that the assembly of other *E. coli* EtBr efflux proteins [20] is also SRP dependent. In an additional control experiment, we observed that arabinose itself did not have any effect on the expression of MdfA-PhoA in wild-type *E. coli* cells (data not shown). Taken together, the drug transport and the alkaline phosphatase activity assays suggest that although MdfA is expressed and localized to the membrane, its proper assembly into an active membrane transporter is defective if Ffh is lacking.

### Association of Ffh with Cytosolic Ribosomes

**Figure 4:**
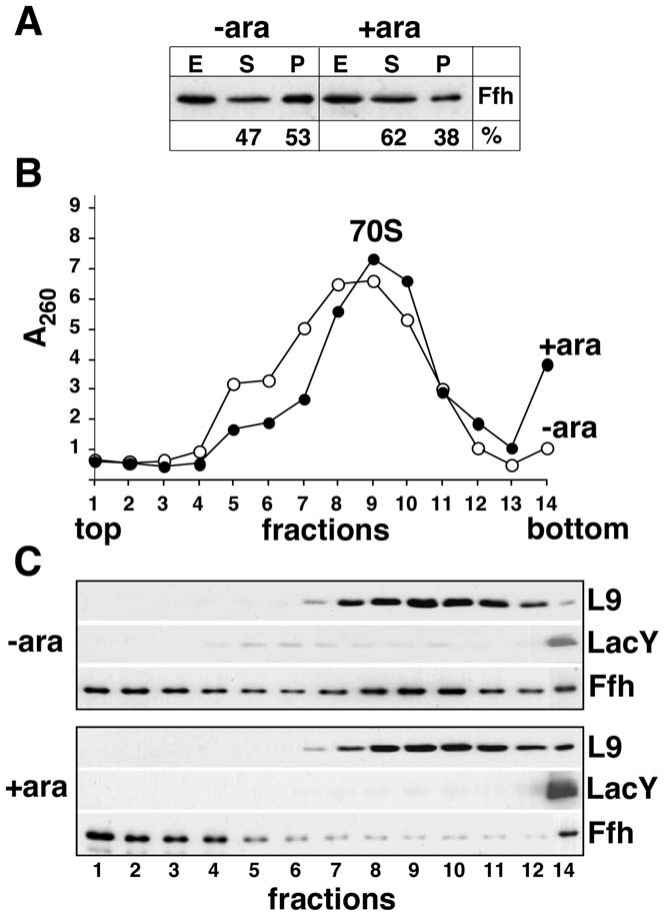
Co-sedimentation of Ffh with 70S ribosome from cells depleted of FtsY. *E. coli* FJP10 harboring pT7-5/LacY were grown for 6.5 h with or without 0.05% arabinose (+/−ara). (A) Cells were fractionated and samples of extracts (E, 10 µg), supernatants (S, 10 µg) and pellets after ultracentrifugation (P, 4.5 µg) were analyzed by Western blotting. The Ffh band was quantified by densitometry. (B, C) The ultracentrifugation pellets (containing cytosolic ribosomes and membranes) were separated by sucrose gradient (see Material and Methods). (B) Optical density at 260 nm of each fraction. (C) Western blotting of each fraction with antibodies against the ribosomal protein L9, LacY, and Ffh.

Earlier, we observed that post-translational proteolysis or mRNA transcription or degradation do not account for the low expression of membrane proteins in FtsY-depleted cells, suggesting that the decreased expression might be due to inhibition of translation. In an attempt to test a possible role for SRP in the reduced expression of membrane proteins in FtsY-depleted cells, we asked whether SRP accumulates on cytosolic ribosomes under these conditions. Extracts prepared from FtsY-depletion cells (Fig. 4A) were separated by sucrose gradient centrifugation. Fractions were collected and examined for ribosomal RNA (A_260_), Ffh, the membrane protein LacY, and the ribosomal protein L9 (Western blots). As observed previously by other means [14], membranes of FtsY-depleted cells contain a very small amount of membrane bound ribosomes and LacY, compared to non-depleted cells (Fig. 4B and C, fraction 14). Figure 4C also shows that cytosolic 70S ribosomes from both depleted and non-depleted samples migrate mainly in fractions 8–11 but the amount of Ffh that co-migrates with the ribosomes is dramatically higher in samples from FtsY-depleted cells compared to non-depleted cells. These results thus revealed a link between the inhibition of membrane protein expression and the association of Ffh with cytosolic ribosomes.

## Discussion

In this work we characterized in a systematic manner the effects of FtsY- or Ffh-depletion on the synthesis, localization, and functional assembly of 2 hybrid versions of the integral membrane protein MdfA. The results show that only cells depleted of FtsY exhibited considerable decrease in MdfA expression, whereas the soluble protein SecA remained unaffected. By studying various stages of membrane protein expression under these conditions, we showed that inhibition of membrane protein expression in FtsY-depleted cells might occur at the level of translation. Understanding the differences between our *in vivo* studies and recent *in vitro* studies, which showed that ribosomes isolated from FtsY-depleted cells are inactive in general [21], may reveal additional regulatory factors that play specific roles in membrane protein translation.

We previously showed that concomitantly with the inhibition of membrane protein expression [7], [14] the amount of membrane-associated ribosomes in FtsY-depleted cells is dramatically reduced [14], suggesting that if membrane proteins are synthesized under these conditions, they must be translated by cytosolic ribosomes. We reasoned that a major regulator of membrane protein translation by cytosolic ribosomes could be the SRP, because of its capacity to bind ribosome-hydrophobic nascent peptide complexes. The observation that inhibition of membrane protein expression under FtsY-depletion conditions coincides with accumulation of Ffh-ribosome complexes in the cytoplasm (Fig. 4), raises the possibility that indeed, SRP might be able to regulate cytosolic ribosomes translating membrane proteins by direct interaction. Therefore, in addition to its role in the proper targeting of ribosomes translating membrane proteins to the SecYEG translocon, we propose that SRP might act on cytosolic ribosomes synthesizing membrane proteins by limiting their translation. Such a regulatory mechanism would be needed under unbalanced physiological situations (such as over-expression) to prevent premature synthesis of membrane proteins in the cytoplasm.

Our studies present the first direct comparison of the biogenesis of model membrane proteins under FtsY- and Ffh-depletion conditions. The results support previous suggestions that Ffh is required for the proper integration and assembly of membrane proteins [5], [6], [8]. Similarly, our results are in agreement with previous indications that FtsY-depletion decreased expression of certain integral membrane proteins [7], [14]. These observations together with the finding that the amount of membrane-bound ribosomes does not decrease in Ffh-depleted cells [14], [22], whereas FtsY-depleted cells have almost no membrane ribosomes (Fig. 4) [14], supports the notion that Ffh and FtsY might play a role in different stages during the pathway of membrane protein biogenesis.

According to a common model of the bacterial SRP pathway [23], Ffh has an essential role in membrane targeting of ribosomes translating membrane proteins. Our current and previous results [14], [22], [24] favor an alternative model of the bacterial SRP pathway, as previously proposed [3]. According to this model, the main role of SRP would be to coordinate the assembly on the translocon, of membrane-bound ribosomes translating membrane proteins. In other words, according to this scenario, SRP may act downstream of the SRP-receptor during the biogenesis of membrane proteins in *E. coli*. Hence, in the absence of SRP, membrane proteins are presumably still translated by membrane bound ribosomes but they do not insert properly into the membrane. Possibly, the improper integration of membrane proteins in Ffh-depleted cells occurs spontaneously or with the assistance of proteins others than the SecYEG translocon. Our current results also suggest the possibility that SRP has an additional quality control role in preventing cytoplasmic expression of membrane proteins when the targeting pathway is blocked. This hypothesis is currently tested by overexpressing the SRP and analyzing its effect on membrane protein biogenesis.

## Materials and Methods

### *E. coli* Strains and Growth Conditions

*E. coli* FJP10 [17] was used for depletion of FtsY. *E. coli* WAM121 [6] was used for depletion of Ffh. Both strains require arabinose (0.05%–0.2%) for growth and induction of FtsY or Ffh. Typically, cells were cultured in LB (*E. coli* WAM121) or 2YT (*E. coli* FJP10) media supplemented with the appropriate inducers and antibiotics (kanamycin, chloramphenicol and spectinomycin were used at 10 µg/ml and ampicillin at 100 µg/ml). MdfA-PhoA hybrids [16] were expressed from the ampicillin resistant plasmid pT7-5 under regulation of the native *mdfA* promoter. For depletion experiments, overnight cultures were centrifuged at room temperature, washed 3 times with plain LB, diluted to 0.02–0.03 A_600_ units and grown with and without arabinose at 37°C.

### Cell Fractionation

Preparation of cell extracts and cell fractionation were performed as described previously [14] with some modifications. To analyze the effect of FtsY- or Ffh-depletion on protein expression, depleted and non-depleted cells were harvested at the indicated time, pelleted by low-speed centrifugation and re-suspended in ice-cold 5% sucrose solution in buffer A (50 mM Tris-HCl pH 8.0, 100 mM NaCl, 1 mM EDTA, 0.1 mM DTT, and 1 mM Pefabloc) to the same cell density (A_600_ ∼10 units). Extracts were prepared by three cycles of brief sonication (10 s at 2 min intervals) on ice, followed by centrifugation (13,000 rpm for 10 min) to remove cell debris. To separate different fractions, cell extracts were subjected first to ultracentrifugation (65 min, 80,000 rpm, 4°C, rotor TLA 100.2) in a tabletop Optima TLX ultracentrifuge (Beckmann) Pellet containing crude membranes, ribosomes and aggregates was re-suspended in 50 µl of the 5% sucrose solution in buffer A and membranes were purified further by floatation centrifugation in a step-wise sucrose gradient in buffer A, as described [14]. The floated membrane ‘ring’ fraction (about 350 µl) was collected, diluted 3 times with buffer A and subjected to ultracentrifugation, as described above. To remove peripherally associated proteins, purified membranes were resuspended in freshly prepared solution of Na_2_CO_3_ (0.2 M, pH 11) and incubated 20 min on ice before ultracentrifugation. Membrane pellet was re-suspended in 1% SDS buffer.

Separation of cytosolic ribosomes and membranes was performed by sucrose gradient cenrifugation. To this end, preparation of cell extracts and the first ultracentrifugation were carried out as described above, but buffer A was replaced for buffer B (20 mM HEPES pH 7.5, 10 mM MgCI_2_, 250 mM NH_4_CI, 5 mM β-mercapto-ethanol and 1 mM Pefabloc). Buffer C (20 mM HEPES pH 7.5, 10 mM MgCI_2_, 500 mM NH_4_CI, 0.2 mM DTT and 0.01% Igepal CA-630) was used for re-suspention of pelleted ribosomes and membranes and for preparation of 7.5%–25% linear sucrose gradient. After centrifugation (60 min, 54,000 rpm, 4°C) in TLS 55 swinging bucket rotor, fractions (105–115 µl) were collected from the top of a sucrose gradient.

### Western Blotting

Protein concentration was measured using a Bradford assay or a modified Lowry procedure [25] in the presence of 2.5% (wt/vol) SDS, using BSA as a standard. Protein samples were incubated for 10 min at 75°C or for 30 min at 37°C with LacY. SDS-PAGE was conducted according to Laemmli [26]. Western blotting was performed as described previously [27] using rabbit affinity-purified antibodies to alkaline phosphatase (Rockland) or to SecA and goat anti-L9 antibodies (lab collection). Affinity-purified antibodies to FtsY and Ffh were prepared in the course of this study, using NTA-purified 6-His tagged proteins. Goat anti-rabbit and donkey anti-goat antibodies conjugated to horseradish peroxidase were used as secondary antibodies. Scanning densitometry was performed with a Bio-Rad Imaging Densitometer (Model GS-690).

### Pulse and Chase Experiments

[^35^S] methionine labeling was performed essentially as described previously [16]. Briefly, *E. coli* FJP10 expressing the MdfA-PhoA hybrids, were grown in 2YT medium for 4.5 h. Cultures were centrifuged at room temperature, washed and re-suspended (to A_420_ of 0.5–1.0) in M9 minimal medium containing 0.4% glycerol and all the amino acids (0.05 mg/ml) except for methionine, and starved for 1 h at the 37°C shaker. [^35^S] methionine was then added (50 µCi/ml, 1000 Ci/mmol, Amersham), and after 2 min of labeling an excess of cold methionine was added (1 mM). Samples (1.8 ml) were transferred, at the indicated time points, into ice-cold tubes containing 0.2 ml of 50% trichloroacetic acid (TCA). After TCA-precipitation pellets were solubilized in buffer A containing 1% SDS (20 min at 37°C), and diluted 10 times with buffer A (containing 0.2% Triton X100). Non-soluble material was removed by centrifugation (table-top microfuge, 13,000 rpm, 10 min), and immunoprecipitation was carried out using antibodies to alkaline phosphatase and Protein A Sepharose, as described previously [16]. After an extensive wash of the Protein A Sepharose beads with buffer D (as buffer A, but with 1M NaCl and 1% Triton X100) and a brief wash with 50 mM Tris-HCl (pH 8.0), the beads were incubated with SDS sample buffer. The eluate was subjected to SDS-PAGE and autoradiography.

### RNA Extraction and Quantitative RT-PCR (qRT-PCR) Amplifications

For extraction of total RNA, 1 ml of culture was mixed with 0.1 ml of ice-cold phenol-ethanol stop solution (5% phenol in ethanol) and cells were collected by centrifugation. RNA was extracted by using the YRB50 kit (RBC bioscience) according to the manufacturer's protocol. Following elution, nucleic acid concentration was determined by spectrophotometry (NanoDrop). Total RNA (1 µg) was reverse-transcribed to cDNA using the ImProm-II RT kit (Promega, Madison, WI) with random hexamer primers according to the manufacturer's instructions. Real-time PCR was done on an ABI 7300 machine (Applied Biosystems, Foster City, CA) with Syber Green PCR mastermix (Applied Biosystems) in 15 µl volume containing 0.01 ng cDNA. For amplification we used the following primers: forward *mdfA*, 5′-cctggtagtccacgccgtaa; reverse *mdfA*, 5′-ctcaagggcacaacctccaa; forward 16S, 5′-cctggtagtccacgccgtaa; reverse 16S, 5′-ctcaagggcacaacctccaa. The expression level of *mdfA* was normalized to the expression level of 16S rRNA.

### Alkaline Phosphatase and Drug Efflux Activities of MdfA-PhoA Hybrids

*E. coli* WAM121 harboring plasmid pT7-5/MdfA379-PhoA was grown for 2.5-3 h in LB without arabinose (Ffh-depletion) and with arabinose (control). The alkaline phosphatase activity in permeabilized cells was estimated by measuring the rate of hydrolysis of p-nitrophenyl phosphate as described previously [16]. The EtBr efflux activity was determined with Ffh-depleted or non-depleted *E. coli* WAM121 carrying plasmid pT7-5/MdfA-PhoA or plain vector (pT7-5) by fluorescence recording test, as described previously [19].
